# *Bursaphelenchus xylophilus* is killed by homologues of 2-(1-undecyloxy)-1-ethanol

**DOI:** 10.1038/srep29300

**Published:** 2016-07-11

**Authors:** Junheon Kim, Sang-Myeong Lee, Chung Gyoo Park

**Affiliations:** 1 Institute of Agriculture and Life Science, Gyeongsang National University, Jinju 52828, Republic of Korea; 2 SM Biovision Co., Jinju 52828, Republic of Korea; 3 Institute of Life Science, Gyeongsang National University, Jinju 52828, Republic of Korea; 4 Division of Applied Life Science (BK21^+^ Program) Gyeongsang National University, Jinju 52828, Republic of Korea

## Abstract

2-(1-Undecyloxy)-1-ethanol, monochamol, is a male-produced aggregation pheromone of the *Monochamus* species, which are efficient vectors of the pine wood nematode (PWN), *Bursaphelenchus xylophilus*, which cause devastating damage to pines worldwide. The nematicidal activity of synthetic monochamol and its homologues (ROEtOH: R = C_7_-C_13_) were investigated to find potential alternatives to the currently used PWN control agents abamectin and emamectin. Compounds with C_7_-C_13_ chain length alkyl groups exhibited 100% nematicidal activity at a concentration of 1000 mg/L. At a concentration of 100 mg/L, 2-(1-nonyloxy)-1-ethanol (C_9_OEtOH), 2-(1-decyloxy)-1-ethanol (C_10_OEtOH), 2-(1-undecyloxy)-1-ethanol (C_11_OEtOH), and 2-(1-dodecyloxy)-1-ethanol (C_12_OEtOH) showed 100% nematicidal activity, but the others showed weaker activities. C_11_OEtOH showed similar nematicidal activity to abamectin in terms of LD_90_ values, which were 13.30 and 12.53 mg/L, respectively. However, C_9_OEtOH, C_10_OEtOH, and C_12_OEtOH (LC_90_ values: 53.63, 38.18, and 46.68 mg/L, respectively) were less effective than C_11_OEtOH and abamectin. These results indicate that monochamol could be an effective alternative agent against PWN. The relationship of insecticidal and nematicidal activity to different carbon chain lengths in compounds is discussed.

Pine wilt disease, caused by the pine wood nematode (PWN; *Bursaphelenchus xylophilus*), is a major threat to global pine forest ecosystems in locations, including Japan, Korea, China, Taiwan, Portugal, and Spain^1^,^2^. The global impact of the presence of the PWN and its likelihood of invading different parts of the world are of increasing economic concern^1^,^2^. Since its first report in Busan city in 1988, it has spread to several other parts of the Korean peninsula^3^,^4^. Most damaged pine trees are red pine (*Pinus densiflora*) and black pine (*P. thunbergii*), and *P. koraiensis* has also recently been identified as a susceptible species in Korea^5^. *P. nigra* and *P. radiate* have also been reported to be susceptible to PWN in Portugal and Spain, respectively^2^,^6^. As *Pinus* species are also a predominant tree species in the Korean forest and are highly susceptible to PWN, the ecological and economic damage is substantial^7^. PWN is predicted to have severe economic consequences for the conifer forestry industry in the EU^8^.

Abamectin and emamectin benzoate, which both belong to the family of avermectins, are primarily used as trunk-injection agents for the control of PWN^9^,^10^,^11^. These agents are known to be effective against PWN^12^ and safe for the environment^13^. However, resistance of nematodes^14^ and insect pests^15^,^16^ to avermectins has been reported, although PWN has not yet been reported to show resistance. As in the case of other groups of insecticides^17^, the continuous use of a single pesticide may induce resistance to those agents in PWN. To avoid the development of resistance and to achieve efficient control of PWN, the alternating use of diverse agents is recommended.

*Monochamus* species are known as efficient vectors of PWN in many countries: *M. alternatus* and *M. saltuarius* in Korea and Japan, *M. carolinensis* in the United States, and *M. galloprovincialis* in Europe^18^. Male adults of *M. galloprovincialis*, *M. carolinensis*, and *M. alternatus* produce an aggregation pheromone, 2-(1-undecyloxy)-1-ethanol (monochamol), that attracts both conspecific females and males^18^,^19^,^20^. *M. saltuarius* is also reported to be attracted to monochamol^21^,^22^.

The nematicidal activity of monochamol was found by chance during our experiments on PWN. We were testing the attractiveness of synthetic monochamol to PWN and found the very interesting phenomenon that PWN was killed in monochamol solutions at very low concentration. We have investigated the nematicidal activity of the monochamol and its homologues to find potential alternatives to currently used PWN control agents. This case is the first known instance of *B. xylophilus* being killed by homologues of 2-(1-undecyloxy)-1-ethanol.

## Results and Discussion

The nematicidal activities of 2-(1-alkyloxy)-1-ethanol homologues, undecanol, and abamectin are shown in Table 1. There was a significant difference in nematicidal activities according to chain length. All 2-(1-alkyloxy)-1-ethanol homologues and undecanol showed 100% mortality at a concentration of 1000 mg/L. 2-(1-Alkyloxy)-1-ethanol homologues with C_9_-C_12_ chain length in the alkyl group and undecanol exhibited 100% mortality even at a concentration of 100 mg/L. However, other homologues with shorter or longer chain lengths, such as C_7_OEtOH, C_8_OEtOH, and C_13_OEtOH, showed less than 40% of mortality at the same concentration. At a concentration of 10 mg/L, only C_11_OEtOH and undecanol showed more than 80% nematicidal activity. Undecanol has been reported to show nematicidal activity against PWN^23^. Our synthetic monochamol also contained 1% undecanol. The mortality of PWN at 100 ppm of C_11_OEtOH which was containing 1 ppm undecanol was higher than that at 1 ppm of undecanol (40%) (Table 1). Therefore, the mortality of C_11_OEtOH was mainly due to C_11_OEtOH itself.

Based on the mortality data (Table 1), LC_50_ and LC_90_ values were calculated to compare the toxicity of 2-(1-alkyloxy)-1-ethanol homologues and undecanol in relation to the number of carbon atoms, along with abamectin, which is in practical use in trunk injection^9^,^10^,^11^. The C_11_OEtOH and undecanol showed significantly higher nematicidal activity (13.30 and 15.78 mg/L, respectively) than the rest in terms of LC_90_ values, with no significant difference from abamectin (12.53 mg/L) (Table 2), followed by C_10_EtOH, C_12_OEtOH and C_9_OEtOH. The homologues C_13_OEtOH, C_8_OEtOH, and C_7_OEtOH showed weaker nematicidal activities than the rest.

The nematicidal activity of the test compounds varied with carbon chain length. Alkanols with C_8_-C_11_ carbon chain length and alkylamine with C_16_ showed significantly higher nematicidal activity against PWN than compounds with shorter or longer carbon chain lengths^23^,^24^. In this study, too, C_11_OEtOH showed higher nematicidal activity than other 1-(2-alkyloxy)-2-ethanol homologues with shorter or longer carbon chains.

The relationship between the structure of aliphatic carboxylic acids and their toxicity to nematodes has been sparsely reported. Among linear carboxylic acids with C_4_-C_10_ carbon atom chains, octanoic acid (C_8_ chain length) was the most toxic to two *Drosophila* species^25^. The authors suggested that its higher toxicity might be linked to its structural characteristics, allowing an easy transfer of the compounds through the insect cuticle. Li *et al*. suggested that the steric hindrance of longer-chain analogs affects the toxicity of aliphatic isothiocyanates^26^. The toxicity of compounds increased when the electron population or electron accessibility (richness) increased^27^. In our study, C_11_OEtOH exerted the most effective nematicidal activity, even though its total length is equivalent to tetradecanol (C_14_OH) which showed weak nematicidal activity in a previous study^23^. The oxy group (-O-) in the structure of C_11_OEtOH possibly contributed to the nematicidal activity. The addition of an oxy group to the structure would increase toxicity by increasing the compound’s transferability through insect cuticles. However, the mechanism of nematicidal activity of some homologues of 2-(1-alkyloxy)-1-ethanol remains unknown.

The different susceptibilities of invertebrates and vertebrates to these compounds provide an acceptable therapeutic ratio for pesticide use. In order to overcome resistance development of a pest against pesticides, the activities of the compounds must have different modes of action^28^. The mode of action of avermectin is related to the GABA-gated chloride channel^29^. Kang *et al*.^30^ estimated the inhibitory activity of C_11_OH against acetylcholinesterase (AChE) and glutathione *S*-transferase (GST) of PWN to elucidate the mode of action^30^. Although C_11_OH showed relatively high nematicidal activity, it showed no or little inhibition activity against AChE and GST of PWN^30^. This result suggests that AChE and GST may not be the targets of C_11_OH. For the safe practical use of C11OEtOH as a nematicide, the mode of action of C_11_OEtOH should be investigated.

It is unclear why the pheromone of the *Monochamus* vector species has strong nematicidal activity against PWN, a parasite of *Pinus* spp. The reasons for PWN to spread through the *Monochamus* vector species that produce an aggregation pheromone that can kill them are worthy of future study.

For a trunk injection agent to be effective against PWN, it is necessary for the injected agent to be translocated throughout the tree at an effective concentration. For this purpose, the agent should have adequate water solubility and diffuse to every part of the tree. C_11_OEtOH showed as effective a nematicidal activity as abamectin, which is in practical use as a trunk injection agent. Although C_11_OEtOH has the advantage of being easily synthesized, it shows very low water solubility. Therefore, to develop C_11_OEtOH as a commercial trunk injection against PWN, a water-soluble preparation of C_11_OEtOH should be formulated.

In conclusion, 2-(1-undecyloxy)-1-ethanol (monochamol) can be used as an effective nematicide against pine wood nematode, and water-soluble monochamol formulations are required along with the identification of an effective concentration (dose) for the control of pine wilt disease. Studying the mechanisms of the nematicidal activity of monochamol is also valuable.

## Materials and Methods

### Collection of pine wood nematodes

PWN was extracted by Baermann funnel method^31^ from chips of PWN-infected pines collected from Jinju, Korea in 2014. The PWN were cultured as described by Park *et al*.^32^ until used for bioassay.

### Chemicals

**Figure 1:**
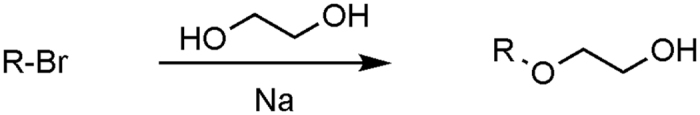
Synthetic scheme of 1-(2-alkyloxyl)-1-ethanol. R is a straight-chain hydrocarbon with 7-13 carbon atoms.

Authentic compounds used for bioassay were synthesized as shown in Fig. 1. Undecanol (99% pure) was obtained from Sigma-Aldrich (St. Louis, MO). Abamectin (1.8% of active ingredient) was purchased from Syngenta (Basel, Switzerland). BFC30, a surfactant, was generously provided by Dongbu Farm Hannon Ltd. (Daejeon, Korea).

### Instrumental analysis

Gas chromatography-mass spectrometry (GC-MS) analysis was performed on a GCMS-QP2010 coupled with a GC2010 (Shimadzu, Kyoto, Japan) equipped with a HP-Innowax (30 m × 0.25 mm i.d., 0.25 μm film thickness; J&W Scientific, Folsom, CA). The oven temperature was programmed as 40 °C for 1 min, then raised to 250 °C at 6 °C/min, and the temperature held for 4 min. Purities of synthesized compounds were checked by GC-FID (GC17A: Shimadzu; DB-5MS: J&W Scientific). ^1^H and ^13^C NMR (500 and 125 MHz, respectively) analysis was performed with a Bruker DRX-500 spectrometer using TMS in CDCl_3_ as an internal standard at Center for Research Facilities of GNU.

### Synthesis of the monochamol and its homologues

2-(1-Undecyloxy)-1-ethanol (C_11_OEtOH) and its homologues were synthesized following the method by Loffredo *et al*. (Fig. 1)^33^. A general procedure followed was; to a 3-necked round-bottom flask, provided with dropping funnel, a reflux condenser, and inlet for nitrogen was added 80 mmole of ethylene glycol (Daejung, Hwasung, Korea). Sodium (23 mmole, Alfa Aesar) was added in small portion to the ethylene glycol with care under vigorous magnetic stirring and the mixture was heated to 60 °C until added sodium dissolved completely. The appropriate 1-bromoalkane (0.20 mmole, Alfa Aesar/Sigma-Aldrich) was added and the solution was then kept heating at 60 °C for 4–6 h. After cooling and adding water, the solution was extracted with diethyl ether three times. The organic layer was washed with 2N HCl and brine, and dried over MgSO_4_. After the solvent was removed, the residue was subjected to silica gel column chromatography to obtain desired compounds (35% diethyl ether in hexane fraction). The NMR data of synthesized compounds (data not shown) were in good agreement with literature values^19^,^33^. The MS data are listed below. NMR data are available in Supplementary Information.

*2-(1-heptyloxy)-1-ethanol* (C_7_OEtOH); Colorless liquid, yield: 72.2%, purity: 99.1%, MS *m/z* (% relative intensity, ion): 129 (0.9, M^+^-CH_2_OH), 115 (0.2), 97 (10.4), 83 (1.0), 70 (10.7), 63 (12.1), 57 (100.0), 45 (45.4), 43 (47.9).

*2-(1-octyloxy)-1-ethanol* (C_8_OEtOH); Colorless liquid, yield: 69.0%, purity: 99.7%, MS *m/z* (% relative intensity, ion): 143 (6.6, M^+^-CH_2_OH), 129 (0.8), 112 (18.6), 111 (45.9), 97 (2.5), 84 (38.5), 83 (22.4), 71 (99.3), 63 (48.6), 57 (100.0), 45 (75.0), 43 (88.5).

*2-(1-nonyloxy)-1-ethanol* (C_9_OEtOH); Colorless liquid, yield: 59.8%, purity: 96.7%, MS *m/z* (% relative intensity, ion): 157 (3.5, M^+^-CH_2_OH), 127 (1.8), 98 (12.5), 85 (37.5), 71 (58.5), 63 (21.2), 57 (60.9), 45 (59.9), 43 (100.0).

*2-(1-decyloxy)-1-ethanol* (C_10_OEtOH); Colorless liquid, yield: 53.5%, purity: 99.4%, MS *m/z* (% relative intensity, ion): 171 (2.5, M^+^-CH_2_OH), 157 (0.2), 140 (5.7), 112 (14.1), 97 (20.0), 85 (65.2), 71 (65.8), 63 (32.2), 57 (100.0), 45 (43.6), 43 (78.8).

*2-(1-undecyloxy)-1-ethanol* (C_11_OEtOH); Colorless liquid, yield: 47.0%, purity: 99.0%, MS (%): 185 (4.3, M^+^-CH_2_OH), 171 (0.3), 154 (6.7), 126 (12.6), 111 (16.0), 97 (39.9), 85 (50.6), 71 (66.6), 63 (28.0), 57 (100.0), 45 (37.5), 43 (59.2).

*2-(1-dodecyloxy)-1-ethanol* (C_12_OEtOH); Colorless liquid, yield: 65.7%, purity: 98.0%, MS *m/z* (% relative intensity, ion): 199 (4.4, M^+^-CH_2_OH), 185 (0.3), 168 (6.3), 140 (12.5), 125 (10.9), 111 (36.8), 97 (47.5), 85 (67.9), 71 (88.4), 63 (46.6), 57 (100.0), 45 (48.9), 43 (82.4).

*2-(1-tridecyloxy)-1-ethanol* (C_13_OEtOH); white solid at 20 °C; yield: 45.9%, purity: 98.2%, MS *m/z* (% relative intensity, ion): 213 (0.6, M^+^-CH_2_OH), 182 (0.7), 154 (1.5), 125 (2.7), 111 (6.1), 97 (12.0), 85 (22.2), 71 (38.7), 63 (22.1), 57 (78.3), 45 (60.3), 43 (100.0).

### Nematicidal activity

Solutions of 2-(1-alkyloxy)-2-ethanol homologues, undecanol, and abamectin (1.8%) were prepared by serial dilution with distilled water containing BFC30 (200 mg/L). The stock solutions were of 1000 ppm, which were then, diluted by 1/10 times in sequence in each step, if a concentration found somewhat effective to the PWN. Undecanol, which was reported to exhibit nematicidal activity^23^ was selected as one of the positive controls because of its 1.0% share in the synthetic monochamol. The stock solution of abamectin (1.8%) was 100 ppm (active ingredient: 18 mg/L) which was also serially diluted. Test solutions (1 mL) were introduced into wells of 24-well plates (Falcon, USA). Each well was then inoculated with about 100 mixed stages (male: female: juvenile = 1:1.2:9.5) of PWN in 10 μL of distilled water. Controls were treated only with BFC30 solutions. The well plates were held under the same conditions as used for nematode colony maintenance. Mortality of nematodes was recorded after 24 h under a stereoscopic microscope. Nematodes were considered as dead if their bodies were straight and when they did not move, even after transferring to clean water. Two to four trials with three to four replicates were performed on different days. Data were pooled for analysis.

### Statistical analyses

Nematode mortality was corrected using Abbott’s formula^34^ and corrected mortality was transformed to arcsine square root values for analysis of variance (ANOVA). Treatment means were compared and separated by Tukey-Kramer HSD test. The LC_50_ value was estimated by probit analysis with dose-response data. Differences in LD_50_ and LD_90_ values between treatments were considered significant if the 95% confidence intervals did not overlap. Statistical analyses were performed using JMP ver. 9.0.2 (SAS Institute Inc., Cary, NC, USA). Mean (±SEM) values of untransformed data are reported.

## Additional Information

**How to cite this article**: Kim, J. *et al*. *Bursaphelenchus xylophilus* is killed by homologues of 2-(1-undecyloxy)-1-ethanol. *Sci. Rep.* **6**, 29300; doi: 10.1038/srep29300 (2016).

## Supplementary Material

Supplementary Information

## Figures and Tables

**Table 1:** Nematicidal activity of 2-(1-alkyloxy)-1-ethanol homologues and undecanol against pine wood nematode, *Bursaphelenchus xylophilus*.

Compound	Corrected mortality (%, mean ± SEM)				
	1000^1^	100	10	1	0.1
C_7_OEtOH	100	25.2 ± 3.4c^2^	8.7 ± 2.8c	–3	–
C_8_OEtOH	100	38.1 ± 3.5b	7.9 ± 1.9bc	–	–
C_9_OEtOH	100	100a	11.3 ± 3.8bc	1.8 ± 0.8b	–
C_10_OEtOH	100	100a	32.3 ± 6.8b	1.1 ± 0.6b	–
C_11_OEtOH	100	100a	83.6 ± 43.3a	45.8 ± 15.9a	2.8 ± 1.4a
C_12_OEtOH	100	100a	17.1 ± 4.3bc	1.7 ± 0.6b	–
C_13_OEtOH	100	7.4 ± 2.6d	–	–	–
Undecanol (C_11_OH)	100	100a	81.7 ± 1.5a	40.0 ± 1.7a	–
Control	0	0d	0c	0c	0a
Statistical values	–	*F*_8,60_ = 621.71 P < 0.001	*F*_7,43_ = 31.27 P < 0.0001	*F*_5,25_ = 9.11, P < 0.0001	*F*_1,6_ = 5.6207 P = 0.0555

^1^mg/L.

^2^Means within a column followed by the same letters are not significantly different (Tukey-Kramer HSD test at *p* = 0.05).

^3^Not tested.

Mortality of control was 3.5 ± 1.0%.

**Table 2:** LC_50_ and LC_90_ values of 2-(1-alkyloxy)-1-ethanol homologues, undecanol and abamectin against pine wood nematode, *Bursaphelenchus xylophilus.*

Compound	LC_50_(mg/L)	95% cl^1^	LC_90_ (mg/L)	95% cl	Slope ± SE	χ^2^ (df^2^)
C_7_OEtOH	133.28	121.92–1459.96f 3	774.75	672.17–905.78c	0.73 ± 0.02	489.91(33)
C_8_OEtOH	101.39	89.31–115.33e	543.02	448.68–676.27c	0.76 ± 0.03	73.54 (14)
C_9_OEtOH	19.35	18.07–20.75d	53.63	48.79–59.47b	1.26 ± 0.04	7514.83(45)
C_10_OEtOH	13.74	12.82–14.80c	38.18	33.42–44.82b	1.25 ± 0.07	292.75 (26)
C_11_OEtOH	1.53	1.37–1.70a	13.30	11.63–15.40a	0.59 ± 0.02	648.93 (42)
C_12_OEtOH	17.11	15.59–18.82d	46.68	41.08–54.00b	1.28 ± 0.06	1835.38 (22)
C_13_OEtOH	231.2	205.20–270.07f	414.61	343.20–534.25c	2.19 ± 0.17	64.04 (29)
Undecanol (C_11_OH)	1.68	1.35–2.04a	15.78	12.21–21.60a	0.57 ± 0.04	7.09 (10)
Abamectin	5.42	4.84–6.06b	12.53	11.04–14.44a	1.53 ± 0.08	7.72 (9)

^1^Confident limit.

^2^Degree of freedom.

^3^The same letters in a column are not significantly different.

## References

[b1] Mota M. M. & Vieira P. R. Pine Wilt Disease: A Worldwide Threat to Forest Ecosystems. (Springer, 2008).

[b2] Inácio M. L. . First detection of *Bursaphelenchus xylophilus* associated with *Pinus nigra* in Portugal and in Europe. For. Pathol. 45, 235–238 (2015).

[b3] Yi C. K., Byun B. H., Park J. D., Yang S. I. & Chang K. H. First finding of the pine wood nematode, *Bursaphelenchus xylophilus* (Steiner et Buhrer) Nickel and its insect vector in Korea. Res. Rep. For. Res. Inst. 38, 141–149 (1989).

[b4] Choi W. I., Koh S. H., Lee S. K. & Choi K. S. Annual Report of Monitoring for Forest Insect Pests and Diseases in Korea: 15-02. (Korea Forest Research Institute, 2015).

[b5] Han H., Chung Y. J. & Shin S. C. First report of pine wilt disease on *Pinus koraiensis* in Korea. Plant Dis. 92, 1251 (2008). 10.1094/PDIS-92-8-1251A 30769465

[b6] Zamora P. . First report of *Bursaphelenchus xylophilus* causing pine wilt disease on *Pinus radiata* in Spain. Plant Dis. 99, 1449 (2015).

[b7] Korea Forest Service. Statistical yearbook of forestry. (Korea Forest Service, 2004).

[b8] Soliman T. . Framework for modelling economic impacts of invasive species, applied to pine wood nematode in Europe. Plos One 7, e45505 (2012). 23029059 10.1371/journal.pone.0045505 PMC3447758

[b9] Kishi Y. The Pine Wood Nematode and the Japanese Pine Sawyer. (Thomas Company Ltd., 1995).

[b10] Korea Forest Service. Guideline for the control of forest diseases and insect pests. (Korea Forest Service, 2008).

[b11] James R., Tisserat N. & Todd T. Prevention of pine wilt of Scots pine (*Pinus sylvestris*) with systemic abamectin injections. Arboric. Urban For. 32, 195–201 (2006).

[b12] Jansson R. K. & Rabatin S. Curative and residual efficacy of injection applications of avermectins for control of plant-parasitic nematodes on banana. J. Nematol. 29, 695–702 (1997). 19274271 PMC2619829

[b13] Saleem M., Hussain D., Ghouse G., Abbas M. & Fisher S. W. Monitoring of insecticide resistance in *Spodoptera litura* (Lepidoptera: Noctuidae) from four districts of Punjab, Pakistan to conventional and new chemistry insecticides. Crop Prot. 79, 177–184 (2015).

[b14] Gopal R. M., Pomroy W. E. & West D. M. Resistance of field isolates of *Trichostrongylus colubriformis* and *Ostertagia circumcincta* to ivermectin. Int. J. Parasitol. 29, 781–786 (1999). 10404276 10.1016/s0020-7519(99)00032-6

[b15] Scott J. G., Roush R. T. & Liu N. Selection of high-level abamectin resistance from field-collected house flies. Musca domestica. Experientia 47, 282–291 (1991). 10.1007/BF01958163 2009941

[b16] Argentine J. A. & Clark J. M. Selection for abamectin resistance in Colorado potato beetle (Coleoptera: Chrysomelidae). Pestic. Sci. 28, 17–24 (1990).

[b17] McKenzie C. L. & Byford R. L. Continuous, alternating, and mixed insecticides affect development of resistance in the horn fly (Diptera: Muscidae). J. Econ. Entomol. 86, 1040–1048 (1993). 8376649 10.1093/jee/86.4.1040

[b18] Akbulut S. & Stamps W. T. Insect vectors of the pinewood nematode: a review of the biology and ecology of *Monochamus* species. For. Pathol. 42, 89–99 (2012).

[b19] Pajares J. A. . Identification and field activity of a male-produced aggregation pheromone in the pine sawyer beetle. Monochamus galloprovincialis. J. Chem. Ecol. 36, 570–583 (2010). 20437083 10.1007/s10886-010-9791-5

[b20] Teale S. A. . A male-produced aggregation pheromone of *Monochamus alternatus* (Coleoptera: Cerambycidae), a major vector of pine wood nematode. J. Econ. Entomol. 104, 1592–1598 (2011). 22066189 10.1603/ec11076

[b21] Ryall K. . Further evidence that monochamol is attractive to *Monochamus* (Coleoptera: Cerambycidae) species, with attraction synergised by host plant volatiles and bark beetle (Coleoptera: Curculionidae) pheromones. Can. Entomol. 147, 564–579 (2015).

[b22] Kim J. . Field evaluation on the synergistic attractiveness of 2-(1-undecyloxy)-1-ethanol and ipsenol to *Monochamus saltuarius*. Entomol. Res. 46, 31–35 (2016).

[b23] Seo S.-M. . Structure-activity relationship of aliphatic compounds for nematicidal activity against pine wood nematode (*Bursaphelenchus xylophilus*). J. Agric. Food Chem. 58, 1823–1827 (2010). 20055406 10.1021/jf902575f

[b24] Nagase A., Kuwahara Y., Tominaga Y. & Sugawara R. Nematicidal activity of akylamine against the pine wood nematode, *Bursaphelenchus lignicolus*. *Agric*. Biol. Chem. 46, 167–172 (1982).

[b25] Legal L., Moulin B. & Jallon J. M. The relation between structures and toxicity of oxygenated aliphatic compounds homologous to the insecticide octanoic acid and the chemotaxis of two species of *Drosophila*. Pestic. Biochem. Physiol. 65, 90–101 (1999).

[b26] Li D. . Synthesis and structure–activity relationships of aliphatic isothiocyanate analogs as antibiotic agents. Med. Chem. Res. 22, 3119–3125 (2013).

[b27] Grodnitzky J. A. & Coats J. R. QSAR evaluation of monoterpenoids’ insecticidal activity. J. Agric. Food Chem. 50, 4576–4580 (2002). 12137478 10.1021/jf0201475

[b28] Bellinger R. G. Pest Resistance to Pesiticides, http://entweb.clemson.edu/pesticid/Issues/pestrest.pdf. Department of Entomology, Clemson University, South Carolina (1996).

[b29] Lasota J. A. & Dybas R. A. Avermectins, a novel class of compounds: Implications for use in arthropod pest control. Annu. Rev. Entomol. 36, 91–117 (1991). 2006872 10.1146/annurev.en.36.010191.000515

[b30] Kang J. S., Moon Y.-S., Lee S. H. & Park I.-K. Inhibition of acetylcholinesterase and glutathione *S*-transferase of the pinewood nematode (*Bursaphelenchus xylophilus*) by aliphatic compounds. Pestic. Biochem. Physiol. 105, 184–188 (2013). 10.1016/j.pestbp.2012.11.007 24238290

[b31] Chawla M. L. & Prasad S. K. Techniques in nematology. II. Comparative efficiency of sampling tools and nematode extraction methods. Ind. J. Nematol. 4, 115–123 (1975).

[b32] Park I.-K. . Nematicidal activity of plant essential oils and components from garlic (*Alliu*m *sativum*) and cinnamon (*Cinnamomum verum*) oils against the pine wood nematode (*Bursaphelenchus xylophilus*). Nematology 7, 767–774 (2005).

[b33] Loffredo C., Pires P. A. R., Imran M. & El Seoud O. A. β-Carotene: A green, inexpensive, and convenient solvatochromic probe for the determination of solvent polarizability. Dyes Pigm. 96, 16–24 (2013).

[b34] Abbott W. S. A method of computing the effectiveness of an insecticide. J. Econ. Entomol. 18, 265–267 (1925).

