# Supplementary material for: Bursaphelenchus xylophilus is killed by homologues of 2-(1-undecyloxy)-1-ethanol
Source: Sci Rep. 2016 Jul 11;6:29300. doi: 10.1038/srep29300 (PMC4941398; doi:10.1038/srep29300)
Supplement: Supplementary Information [file srep29300-s1.pdf]

Supplementary Information to:

Bursaphelenchus xylophilus **is killed by homologues of 2-(1-undecyloxy)-1-ethanol**

Junheon Kim[1,†], Sang-Myeong Lee[4,†], Chung Gyoo Park[1,2,3,*]

[1]Institute of Agriculture and Life Science and [2]Institute of Life Science, [3]Division of Applied Life Science (BK21[+] Program) Gyeongsang National University, Jinju 52828, Republic of Korea, [4]SM Biovision Co., Jinju 52828, Republic of Korea

[†]These authors contributed equally to this study.

[*]Corresponding author: Chung Gyoo Park,

Institute of Life Science, Gyeonsang National University, Jinju, 52828, Republic of Korea

E-mail: parkcg@gnu.ac.kr; Telephone: +827721925; Fax: +827721929

2-(1-heptyloxy)-1-ethanol (C_7OEtOH) (Figure S1);
^{1}H NMR (500 MHz, $CDCl_3$) 3.73 (2H, q-like, J=5), 3.53 (2H, t-like, J=5), 3.47 (2H, t, J=7), 1.58 (2H, quin, J=7), 1.62-1.26 (8H, m), 0.88 (3H, t, J=7);
^{13}C NMR (126 MHz, $CDCl_3$) δ 71.70, 71.44, 61.87, 31.81, 29.67, 29.15, 26.08, 22.61, 14.08.

2-(1-octyloxy)-1-ethanol (C_8OEtOH) (Figure S2);
^{1}H NMR (500 MHz, $CDCl_3$) δ 3.73 (2H, q-like, J=5), 3.53 (2H, t-like, J=5), 3.47 (2H, t, *J* = 6.7 Hz), 1.58 (2H,quin, J=7), 1.39 – 1.22 (m, 10H), 0.88 (3H, t, J=7);
^{13}C NMR (126 MHz, $CDCl_3$) δ 71.69, 71.44, 61.88, 31.82, 29.67, 29.45, 29.26, 26.12, 22.66, 14.09.

2-(1-nonyloxy)-1-ethanol (C_9OEtOH) (Figure S3);
^{1}H NMR (500 MHz, $CDCl_3$) δ 3.73 (2H, q-like, J=5), 3.53 (2H, t-like, J=5), 3.47 (2H, t, *J* = 6.7 Hz), 1.59 (2H,quin, J=7), 1.39 – 1.19 (m, 12H), 0.88 (3H, t, J=7);
^{13}C NMR (126 MHz, $CDCl_3$) δ 71.68, 71.44, 61.88, 31.88, 29.67, 29.56, 29.49, 29.27, 26.12, 22.67, 14.11.

2-(1-decyloxy)-1-ethanol (C_{10}OEtOH) (Figure S4);
^{1}H NMR (500 MHz, $CDCl_3$) δ 3.73 (2H, q-like, J=5), 3.53 (2H, t-like, J=5), 3.47 (2H, t, *J* = 6.7 Hz), 1.59 (2H,quin, J=7), 1.38 – 1.18 (m, 14H), 0.88 (3H, t, J=7);
^{13}C NMR (126 MHz, $CDCl_3$) δ 71.69, 71.45, 61.88, 31.90, 29.67, 29.60, 29.57, 29.49, 29.32, 26.12, 22.69, 14.11.

2-(1-undecyloxy)-1-ethanol (C_{11}OEtOH) (Figure S5);
^{1}H NMR (500 MHz, $CDCl_3$) δ δ 3.73 (2H, q-like, J=5), 3.53 (2H, t-like, J=5), 3.47 (2H, t, *J* = 6.7 Hz), 1.59 (2H,quin, J=7), 1.43 – 1.18 (m, 16H), 0.90 (3H, t, J=6.9);
^{13}C NMR (126 MHz, $CDCl_3$) δ 71.69, 71.45, 61.88, 31.91, 29.67, 29.62 (x 2), 29.60, 29.49, 29.34, 26.12, 22.69, 14.12.

2-(1-dodecyloxy)-1-ethanol (C_{12}OEtOH) (Figure S6);;
^{1}H NMR (500 MHz, $CDCl_3$) δ 3.73 (2H, q-like, J=5), 3.53 (2H, t-like, J=5), 3.47 (2H, t, *J* = 6.7 Hz), 1.59 (2H,quin, J=7), 1.41 – 1.21 (m, 18H), 0.90 (3H, t, J=6.9);
^{13}C NMR (126 MHz, $CDCl_3$) δ 71.68, 71.45, 61.89, 31.92, 29.67 (x2), 29.64, 29.62, 29.60, 29.49, 29.36, 26.13, 22.69, 14.12.

2-(1-tridecyloxy)-1-ethanol (C_{13}OEtOH) (Figure S7);
^{1}H NMR (500 MHz, $CDCl_3$) δ 3.73 (2H, q-like, J=5), 3.53 (2H, t-like, J=5), 3.47 (2H, t, *J* = 6.7 Hz), 1.59 (2H,quin, J=7), 1.37 – 1.20 (m, 20H), 0.88 (3H, t, J=6.9)
^{13}C NMR (126 MHz, $CDCl_3$) δ 71.68, 71.45, 61.88, 31.93, 29.683, 29.675, 29.67, 29.65, 29.62, 29.60, 29.50, 29.36, 26.13, 22.70, 14.12.

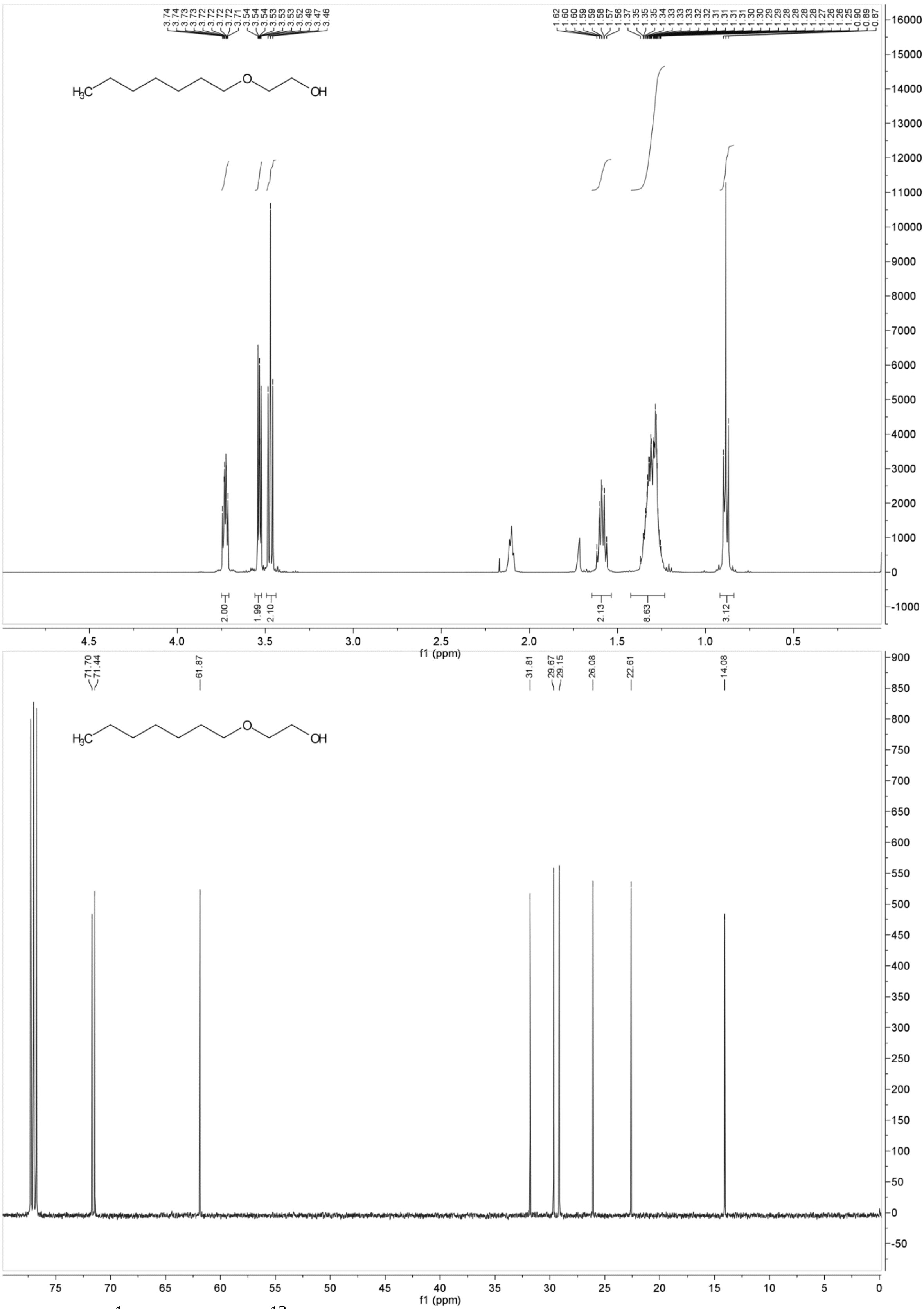

Figure S1. ^{1}H (upper) and ^{13}C (lower) NMR spectrum of *2*-(1-heptyloxy)-1-ethanol (C_7OEtOH).

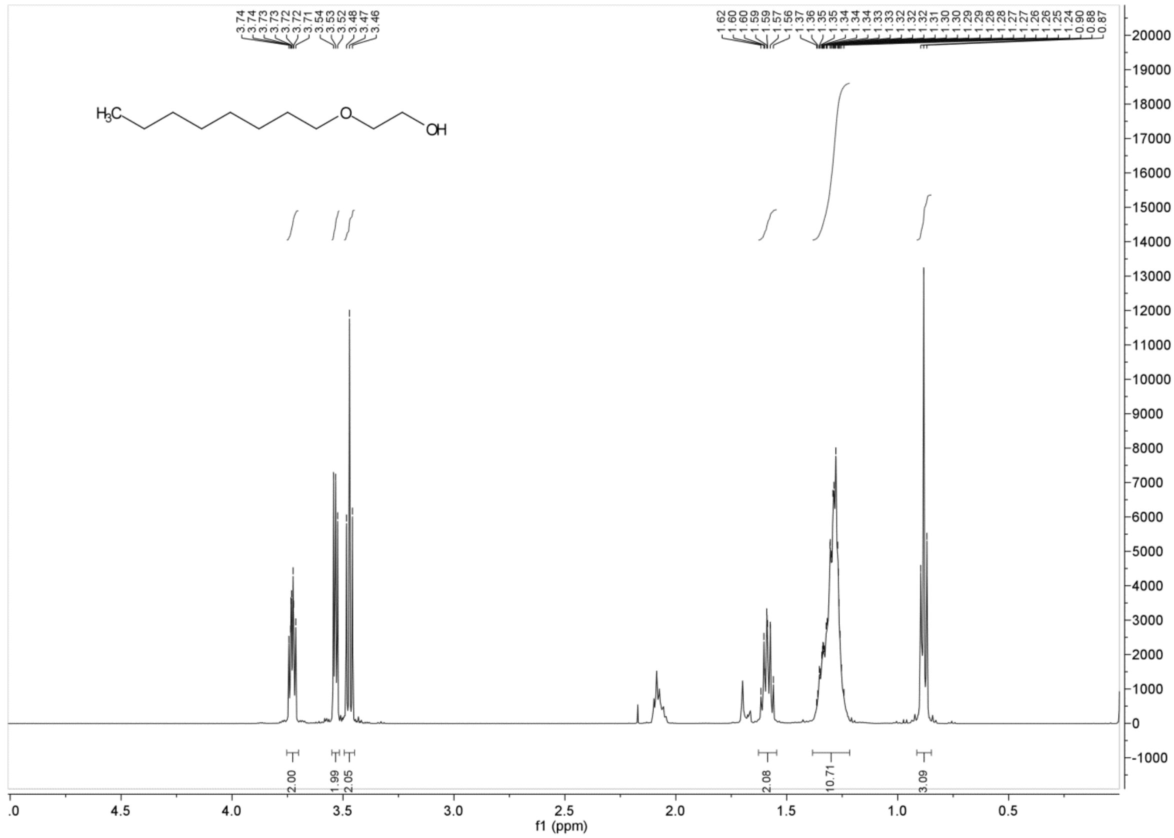

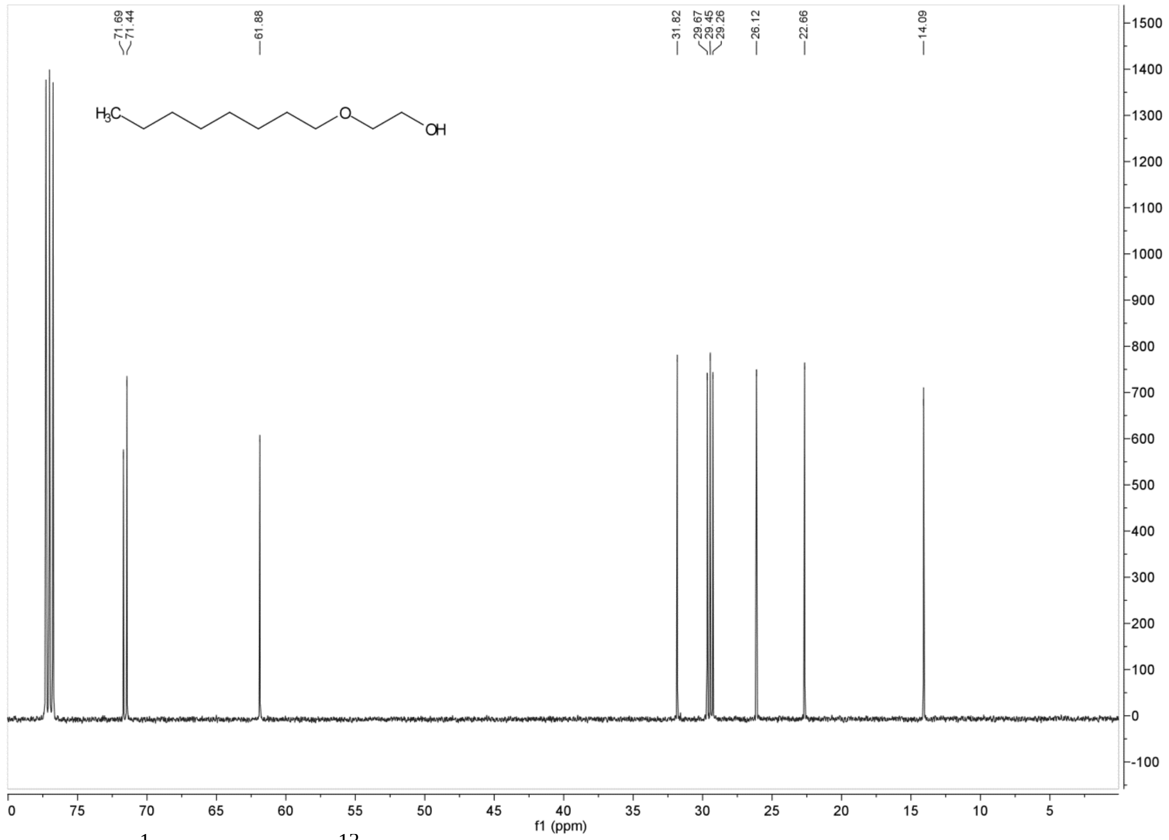

Figure S2. ^{1}H (upper) and ^{13}C (lower) NMR spectrum of *2*-(1-octyloxy)-1-ethanol (C_8OEtOH).

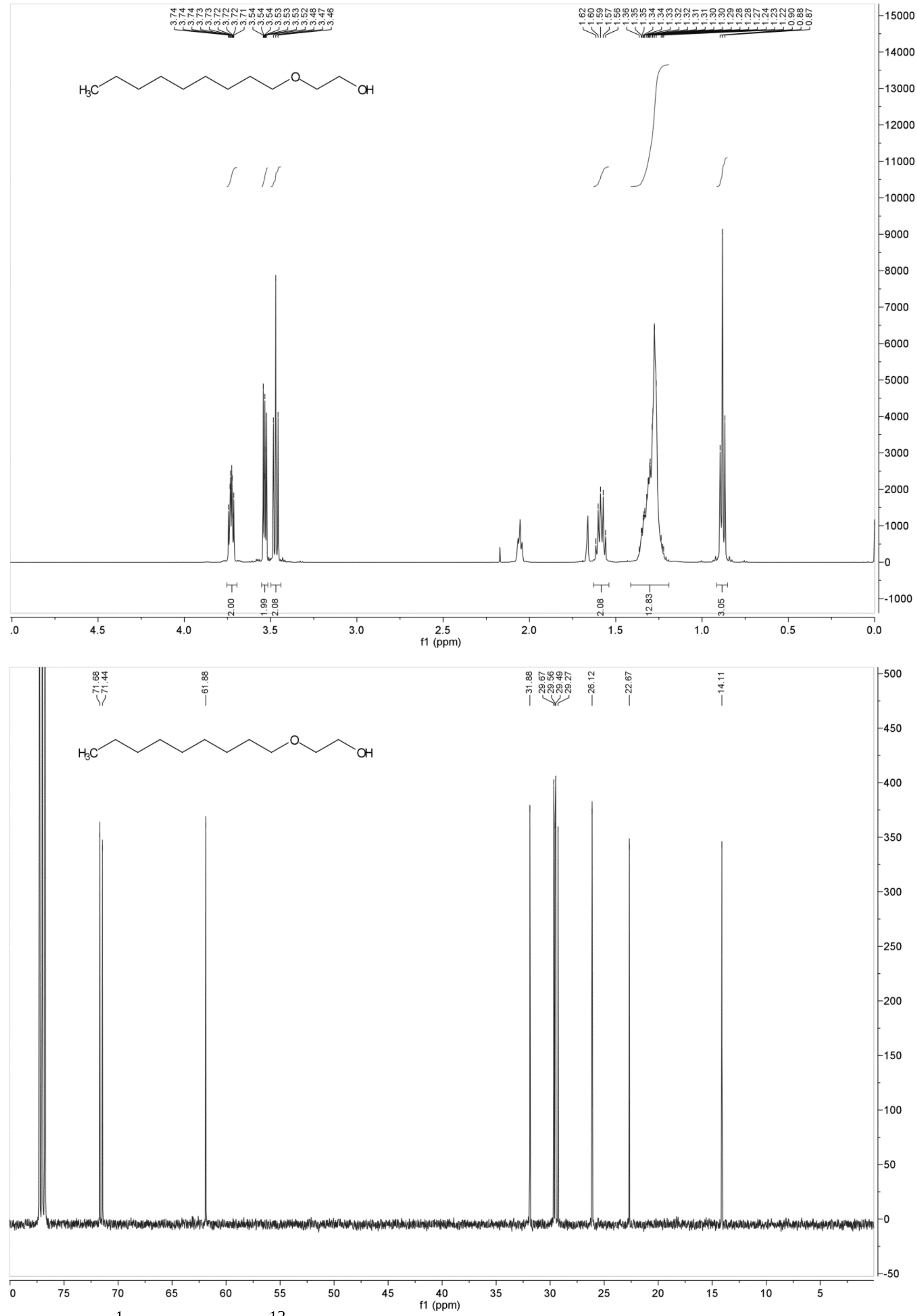

Figure S3. ^{1}H (upper) and ^{13}C (lower) NMR spectrum of *2*-(1-nonyloxy)-1-ethanol (C_9OEtOH).

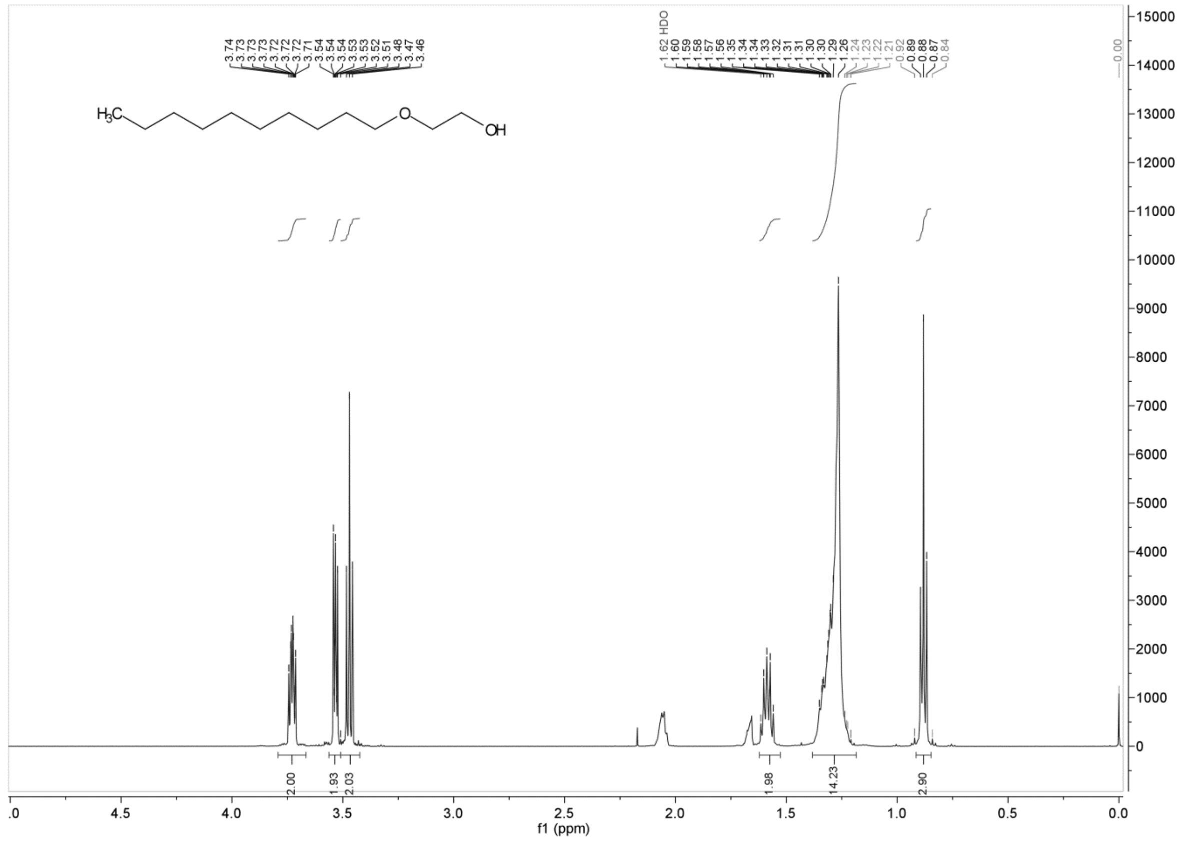

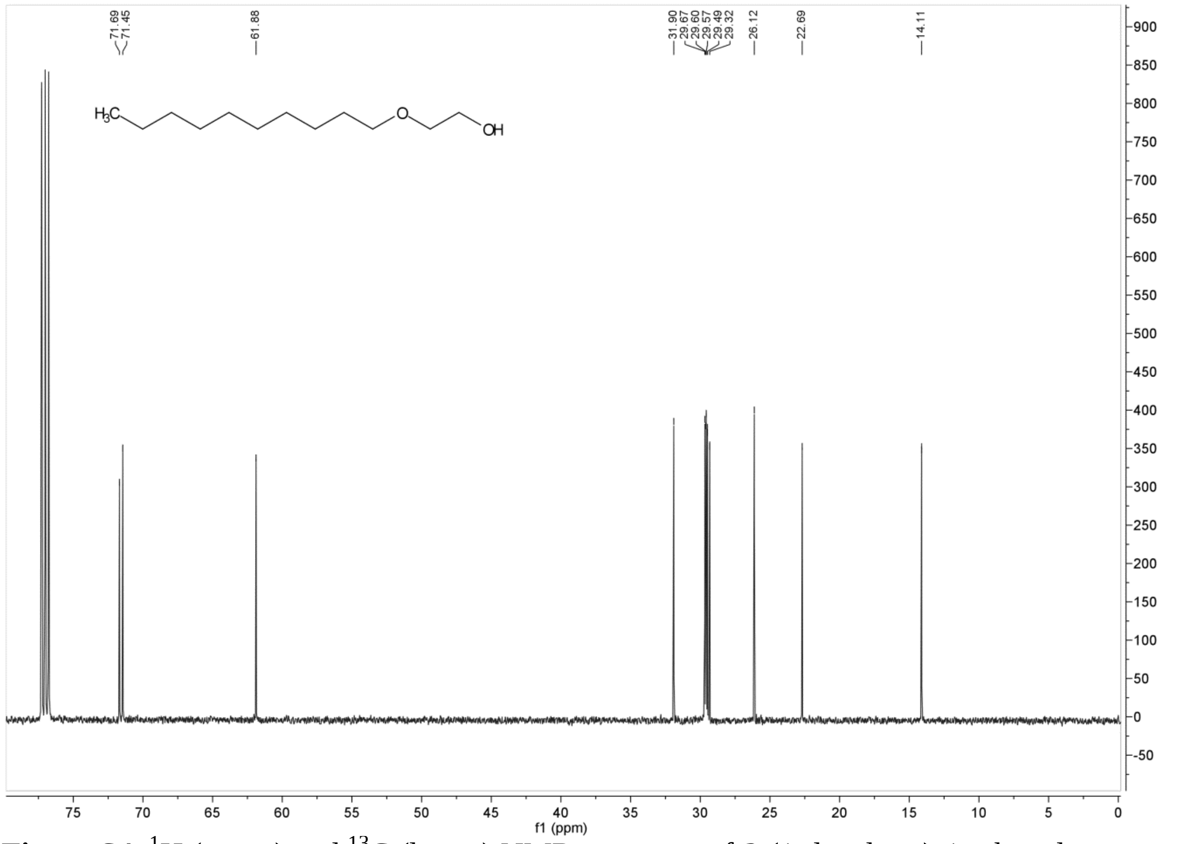

Figure S4. ^{1}H (upper) and ^{13}C (lower) NMR spectrum of *2*-(1-decyloxy)-1-ethanol ($C_{10}OEtOH$).

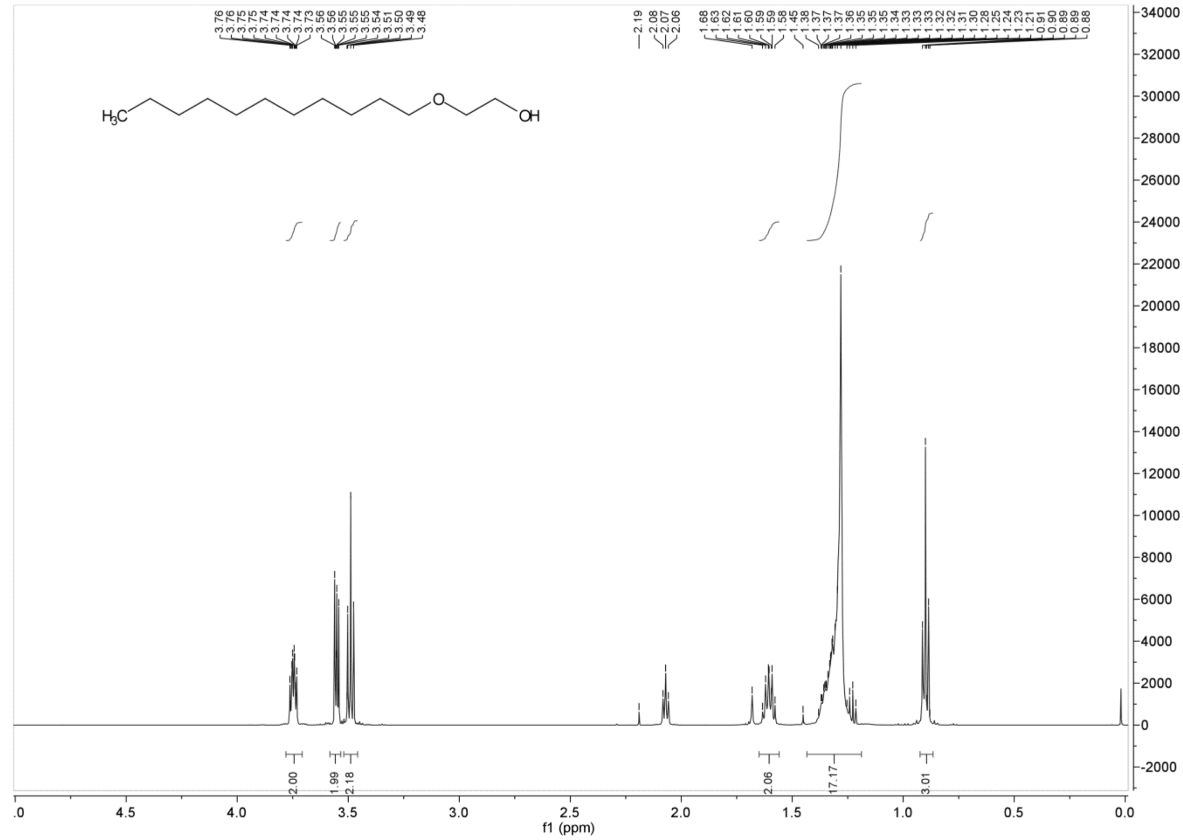

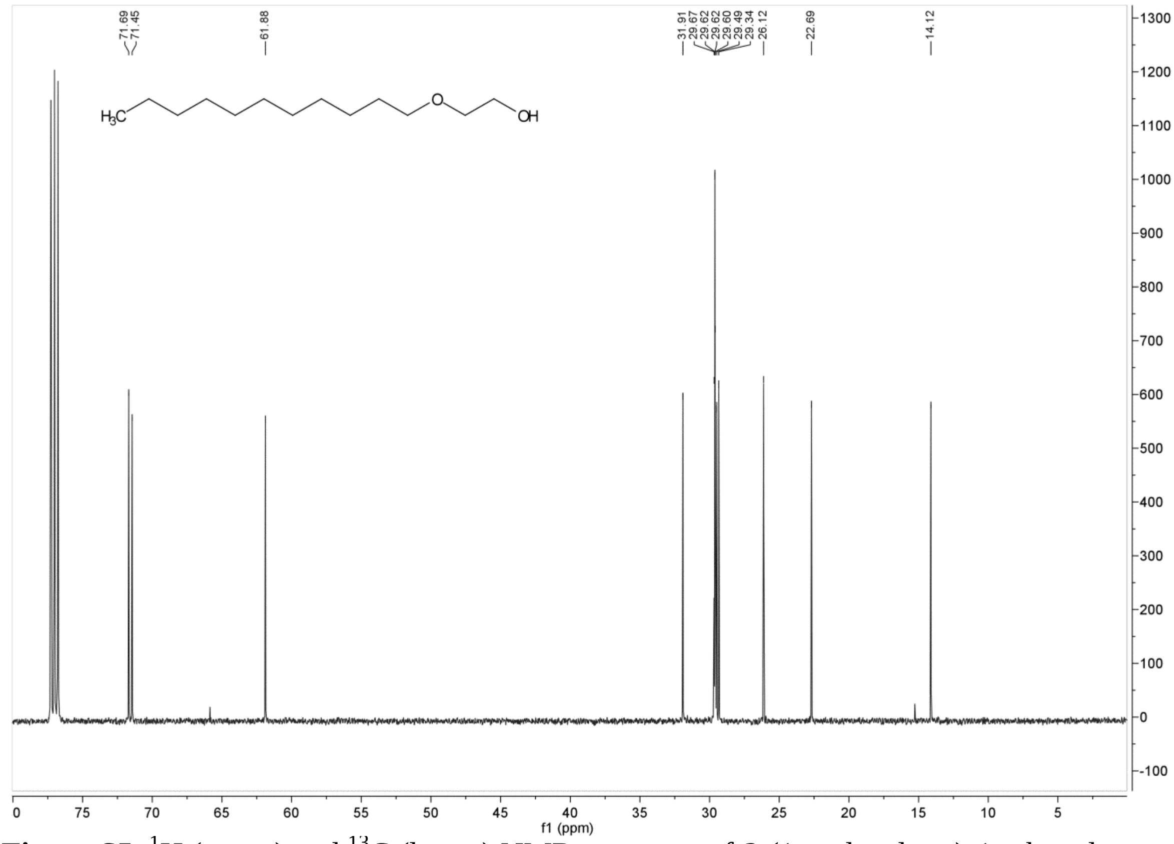

Figure S5. ${}^{1}H$ (upper) and ${}^{13}C$ (lower) NMR spectrum of *2*-(1-undecyloxy)-1-ethanol ($C_{11}OEtOH$).

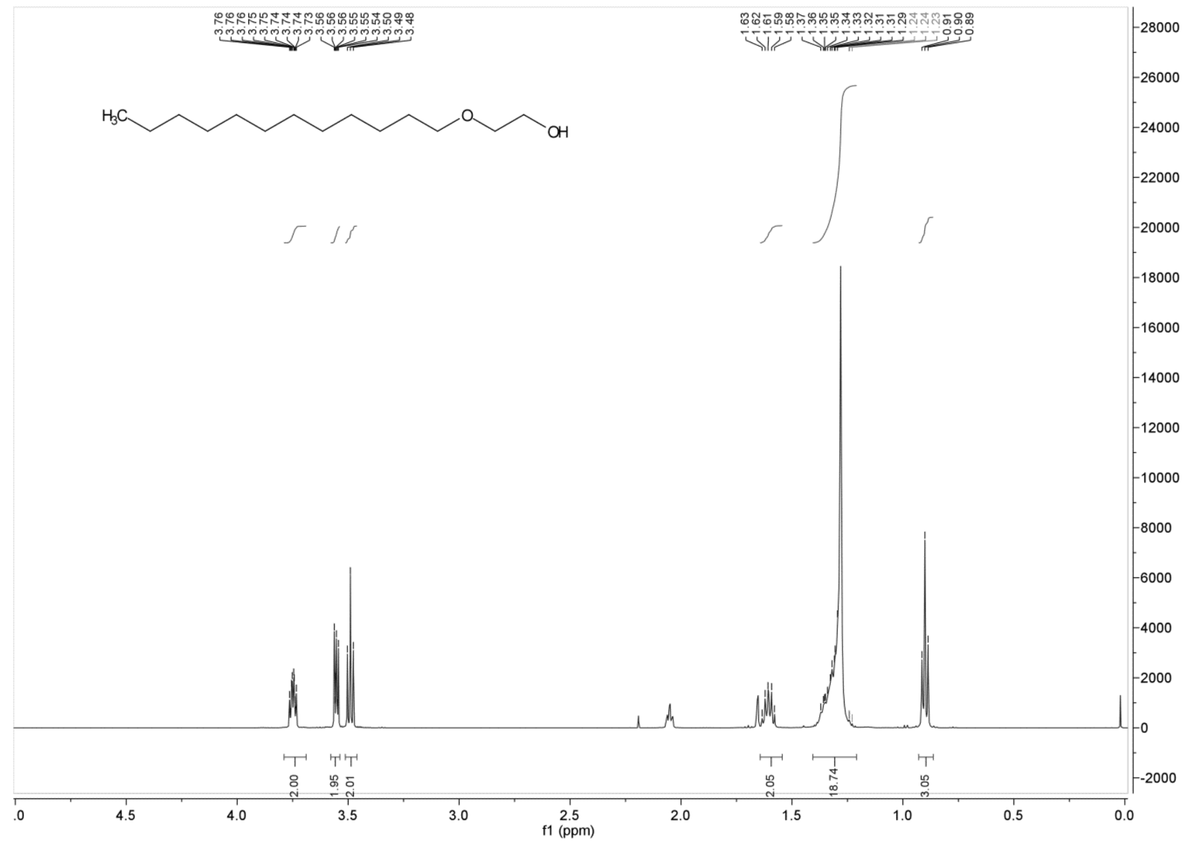

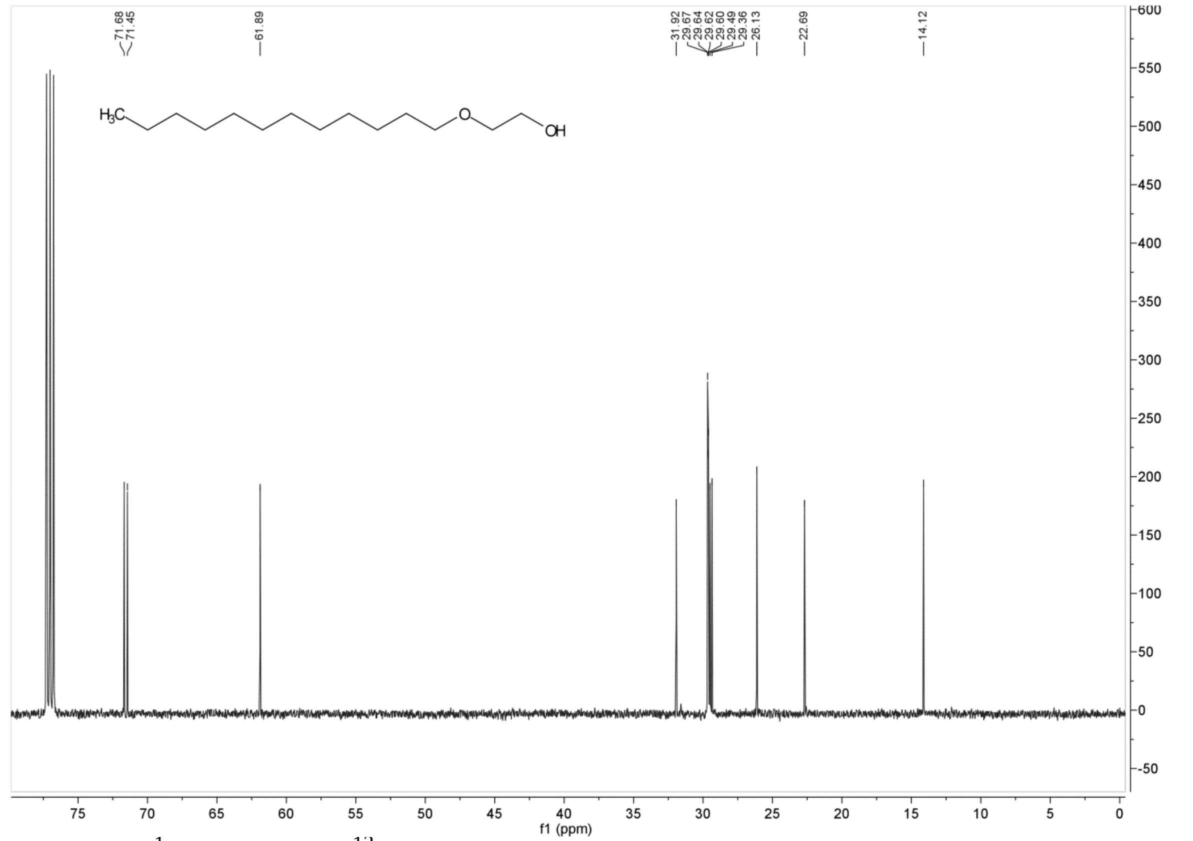

Figure S6. ^{1}H (upper) and ^{13}C (lower) NMR spectrum of *2*-(1-dodecyloxy)-1-ethanol (C_{12}OEtOH).

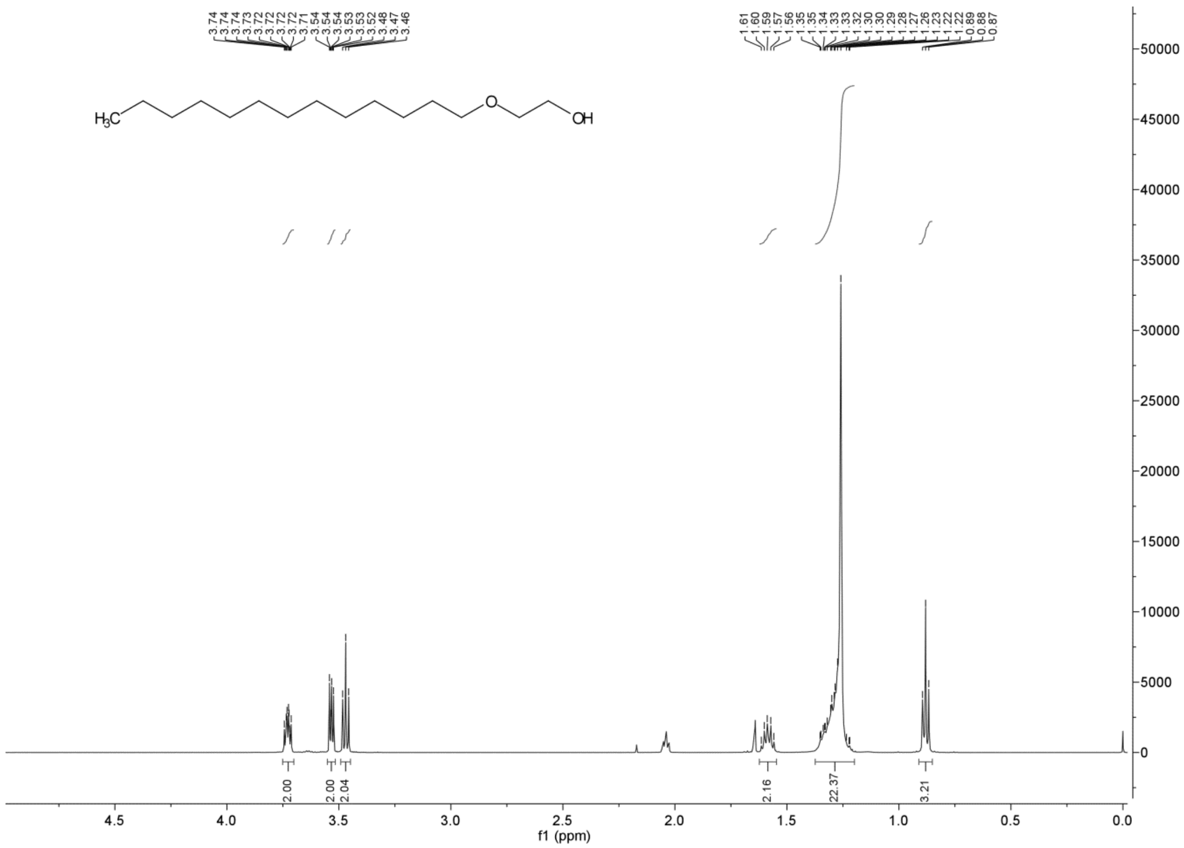

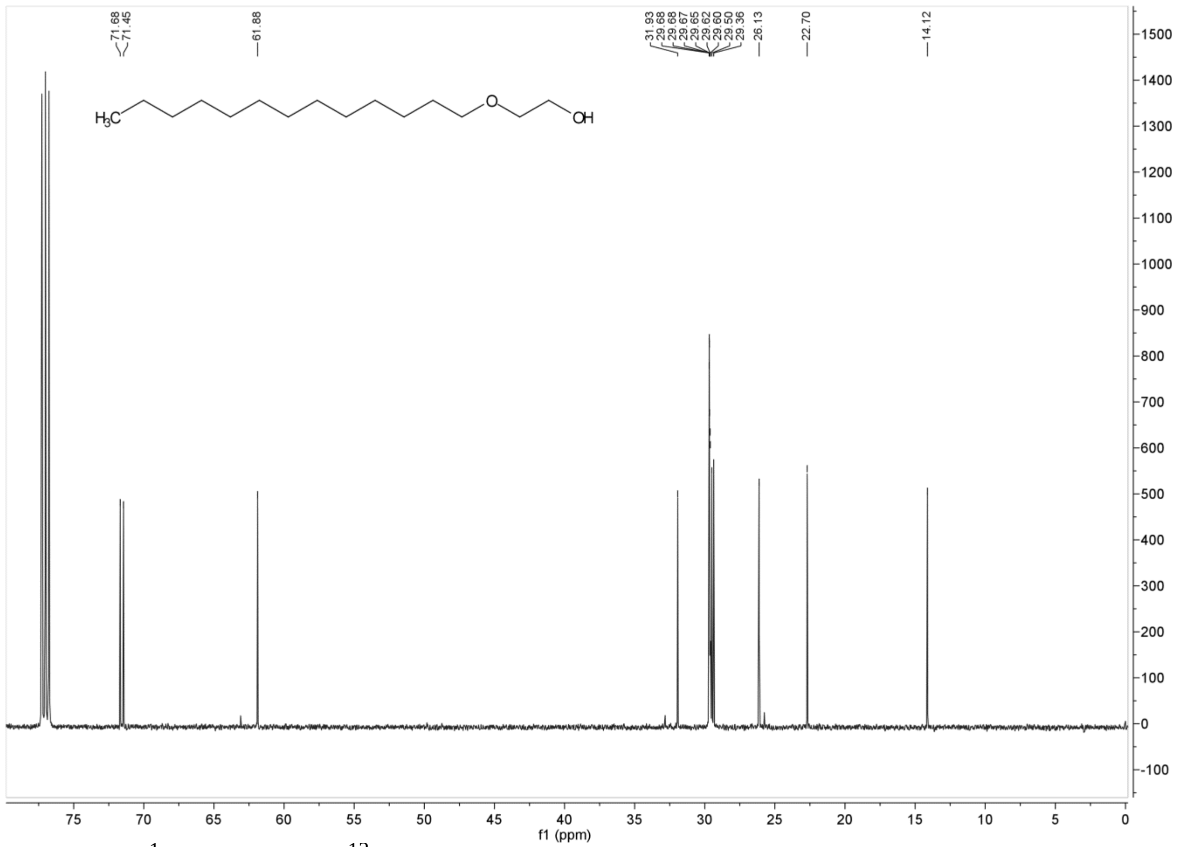

Figure S7. ^{1}H (upper) and ^{13}C (lower) NMR spectrum of *2*-(1-tridecyloxy)-1-ethanol ($C_{13}OEtOH$).
